# Supplementary material for: Effectiveness and Safety of Ceftriaxone Compared to Standard of Care for Treatment of Bloodstream Infections Due to Methicillin-Susceptible Staphylococcus aureus: A Systematic Review and Meta-Analysis
Source: Antibiotics (Basel). 2022 Mar 10;11(3):375. doi: 10.3390/antibiotics11030375 (PMC8944781; doi:10.3390/antibiotics11030375)
Supplement: Supplementary file 1 [file antibiotics-11-00375-s001.zip › antibiotics-1611289-supplementary.pdf]

Table S1: PRISMA checklist.

Section and Topic	Item #	Checklist item	Location where item is reported
**TITLE**			
Title	1	Identify the report as a systematic review.	1
**ABSTRACT**			
Abstract	2	See the PRISMA 2020 for Abstracts checklist.	2
**INTRODUCTION**			
Rationale	3	Describe the rationale for the review in the context of existing knowledge.	2
Objectives	4	Provide an explicit statement of the objective(s) or question(s) the review addresses.	2
**METHODS**			
Eligibility criteria	5	Specify the inclusion and exclusion criteria for the review and how studies were grouped for the syntheses.	2-3
Information sources	6	Specify all databases, registers, websites, organisations, reference lists and other sources searched or consulted to identify studies. Specify the date when each source was last searched or consulted.	2
Search strategy	7	Present the full search strategies for all databases, registers and websites, including any filters and limits used.	2
Selection process	8	Specify the methods used to decide whether a study met the inclusion criteria of the review, including how many reviewers screened each record and each report retrieved, whether they worked independently, and if applicable, details of automation tools used in the process.	2
Data collection process	9	Specify the methods used to collect data from reports, including how many reviewers collected data from each report, whether they worked independently, any processes for obtaining or confirming data from study investigators, and if applicable, details of automation tools used in the process.	3
Data items	10a	List and define all outcomes for which data were sought. Specify whether all results that were compatible with each outcome domain in each study were sought (e.g. for all measures, time points, analyses), and if not, the methods used to decide which results to collect.	2-3
	10b	List and define all other variables for which data were sought (e.g. participant and intervention characteristics, funding sources). Describe any assumptions made about any missing or unclear information.	2-3
Study risk of bias assessment	11	Specify the methods used to assess risk of bias in the included studies, including details of the tool(s) used, how many reviewers assessed each study and whether they worked independently, and if applicable, details of automation tools used in the process.	3
Effect measures	12	Specify for each outcome the effect measure(s) (e.g. risk ratio, mean difference) used in the synthesis or presentation of results.	3
Synthesis methods	13a	Describe the processes used to decide which studies were eligible for each synthesis (e.g. tabulating the study intervention characteristics and comparing against the planned groups for each synthesis (item #5)).	2-3
	13b	Describe any methods required to prepare the data for presentation or synthesis, such as handling of missing summary statistics, or data conversions.	3
	13c	Describe any methods used to tabulate or visually display results of individual studies and syntheses.	3
	13d	Describe any methods used to synthesize results and provide a rationale for the choice(s). If meta-analysis was performed, describe the model(s), method(s) to identify the presence and extent of statistical heterogeneity, and software package(s) used.	3
	13e	Describe any methods used to explore possible causes of heterogeneity among study results (e.g. subgroup analysis, meta-regression).	3
	13f	Describe any sensitivity analyses conducted to assess robustness of the synthesized results.	4
Reporting bias assessment	14	Describe any methods used to assess risk of bias due to missing results in a synthesis (arising from reporting biases).	3
Certainty assessment	15	Describe any methods used to assess certainty (or confidence) in the body of evidence for an outcome.	3
**RESULTS**			
Study selection	16a	Describe the results of the search and selection process, from the number of records identified in the search to the number of studies included in the	4

Section and Topic	Item #	Checklist item	Location where item is reported
		review, ideally using a flow diagram.	
	16b	Cite studies that might appear to meet the inclusion criteria, but which were excluded, and explain why they were excluded.	4
Study characteristics	17	Cite each included study and present its characteristics.	4
Risk of bias in studies	18	Present assessments of risk of bias for each included study.	4
Results of individual studies	19	For all outcomes, present, for each study: (a) summary statistics for each group (where appropriate) and (b) an effect estimate and its precision (e.g. confidence/credible interval), ideally using structured tables or plots.	6-12
Results of syntheses	20a	For each synthesis, briefly summarise the characteristics and risk of bias among contributing studies.	6-7
	20b	Present results of all statistical syntheses conducted. If meta-analysis was done, present for each the summary estimate and its precision (e.g. confidence/credible interval) and measures of statistical heterogeneity. If comparing groups, describe the direction of the effect.	6-12
	20c	Present results of all investigations of possible causes of heterogeneity among study results.	6-12
	20d	Present results of all sensitivity analyses conducted to assess the robustness of the synthesized results.	6-12
Reporting biases	21	Present assessments of risk of bias due to missing results (arising from reporting biases) for each synthesis assessed.	12
Certainty of evidence	22	Present assessments of certainty (or confidence) in the body of evidence for each outcome assessed.	6-12
**DISCUSSION**			
Discussion	23a	Provide a general interpretation of the results in the context of other evidence.	12
	23b	Discuss any limitations of the evidence included in the review.	13
	23c	Discuss any limitations of the review processes used.	13
	23d	Discuss implications of the results for practice, policy, and future research.	13
**OTHER INFORMATION**			
Registration and protocol	24a	Provide registration information for the review, including register name and registration number, or state that the review was not registered.	14
	24b	Indicate where the review protocol can be accessed, or state that a protocol was not prepared.	N/A
	24c	Describe and explain any amendments to information provided at registration or in the protocol.	N/A
Support	25	Describe sources of financial or non-financial support for the review, and the role of the funders or sponsors in the review.	14
Competing interests	26	Declare any competing interests of review authors.	14
Availability of data, code and other materials	27	Report which of the following are publicly available and where they can be found: template data collection forms; data extracted from included studies; data used for all analyses; analytic code; any other materials used in the review.	N/A

Table S2: MOOSE checklist.

Reporting Criteria	Reported (Yes/No)	Reported on Page No.
**Reporting of Background**		
Problem definition	Yes	2
Hypothesis statement	Yes	2
Description of Study Outcome(s)	Yes	2-3
Type of exposure or intervention used	Yes	2-3
Type of study design used	Yes	2-3
Study population	Yes	2-3
**Reporting of Search Strategy**		
Qualifications of searchers (eg, librarians and investigators)	No	N/A
Search strategy, including time period included in the synthesis and keywords	Yes	2
Effort to include all available studies, including contact with authors	Yes	2-3
Databases and registries searched	Yes	2
Search software used, name and version, including special features used (eg, explosion)	Yes	3
Use of hand searching (eg, reference lists of obtained articles)	Yes	2
List of citations located and those excluded, including justification	Yes	4
Method for addressing articles published in languages other than English	Yes	2-3
Method of handling abstracts and unpublished studies	Yes	2-3
Description of any contact with authors	No	N/A
**Reporting of Methods**		
Description of relevance or appropriateness of studies assembled for assessing the hypothesis to be tested	Yes	2 &12
Rationale for the selection and coding of data (eg, sound clinical principles or convenience)	Yes	2
Documentation of how data were classified and coded (eg, multiple raters, blinding, and interrater reliability)	Yes	2-3
Assessment of confounding (eg, comparability of cases and controls in studies where appropriate	Yes	3

Reporting Criteria	Reported (Yes/No)	Reported on Page No.
Assessment of study quality, including blinding of quality assessors; stratification or regression on possible predictors of study results	Yes	3
Assessment of heterogeneity	Yes	3
Description of statistical methods (eg, complete description of fixed or random effects models, justification of whether the chosen models account for predictors of study results, dose-response models, or cumulative meta-analysis) in sufficient detail to be replicated	Yes	3
Provision of appropriate tables and graphics	No	N/A
**Reporting of Results**		
Table giving descriptive information for each study included	Yes	6-7
Results of sensitivity testing (eg, subgroup analysis)	Yes	8
Indication of statistical uncertainty of findings	No	
**Reporting of Discussion**		
Quantitative assessment of bias (eg, publication bias)	Yes	4 & 6-7
Justification for exclusion (eg, exclusion of non–English-language citations)	Yes	2-3
Assessment of quality of included studies	Yes	3
**Reporting of Conclusions**		
Consideration of alternative explanations for observed results	Yes	12
Generalization of the conclusions (ie, appropriate for the data presented and within the domain of the literature review)	Yes	12-13
Guidelines for future research	Yes	13
Disclosure of funding source	Yes	14

Table S3: Search criteria.

((("Ceftriaxone"[Mesh]) OR ((((Ceftriaxone[Text Word]) OR (Rocephin[Text Word])) OR (CTX[Text Word])) OR (CRO[Text Word]))) AND ((("Sepsis"[Mesh]) OR "Bacteremia"[Mesh]) OR (((Bacteremia[Text Word]) OR (BSI[Text Word])) OR (Bloodstream infection[Text Word]))) AND (("Staphylococcal Infections"[Mesh]) OR ((((Methicillin sensitive staphylococcus aureus[Text Word]) OR (methicillin susceptible staphylococcal aureus[Text Word])) OR (Staphylococcal infection[Text Word])) OR (MSSA[Text Word]))))
The following search was performed in PubMed, Embase, and Cochrane Library with keywords and medical subject headings. When searching Embase a filter was used to keep only Embase publications. We searched the ID Week 2021 conference since there is a lag time between abstract presentation in the conference and abstract publication in literature databases.

Table S4: Newcastle–Ottawa Scale (NOS) for observational studies.

	Selection				Comparability		Outcome			
Reference	Representativeness of the exposed cohort	Selection of the non-exposed cohort	Ascertainment of exposure	Demonstration that outcome of interest was not present at start of study	study controls for important factor	study controls for any additional factor	Assessment of outcome	Was follow-up long enough for outcomes to occur	Adequacy of follow up of cohorts	Total score out of 9
Paul, M (2010)	*	*	*	*	*	*	*	*	*	9
Patel, UC (2014)	*	*	*	*	*	*	*	*	*	9
Carr et al 2018	*	*	*	*	*	–	*	*	*	8
Hamad, Y (2020)	*	*	*	*	*	*	*	*	*	9
Barber, KE (2021)	*	*	*	*	*	–	*	*	*	8
Snawerdt, J (2019)	*	*	*	*	–	–	*	*	*	7
Diamante, O (2014)	*	*	*	*	–	–	*	*	*	7
Hamad, Y (2021)	*	*	*	*	*	*	*	*	*	9
Wynn, M (2005)	*	*	*	*	–	–	*	*	*	7
Falsetta, K (2017)	*	*	*	*	–	–	*	*	*	7
Mohamed, A (2020)	*	*	*	*	–	–	*	*	*	7
Bhavan, K (2018)	*	*	*	*	–	–	–	–	*	5

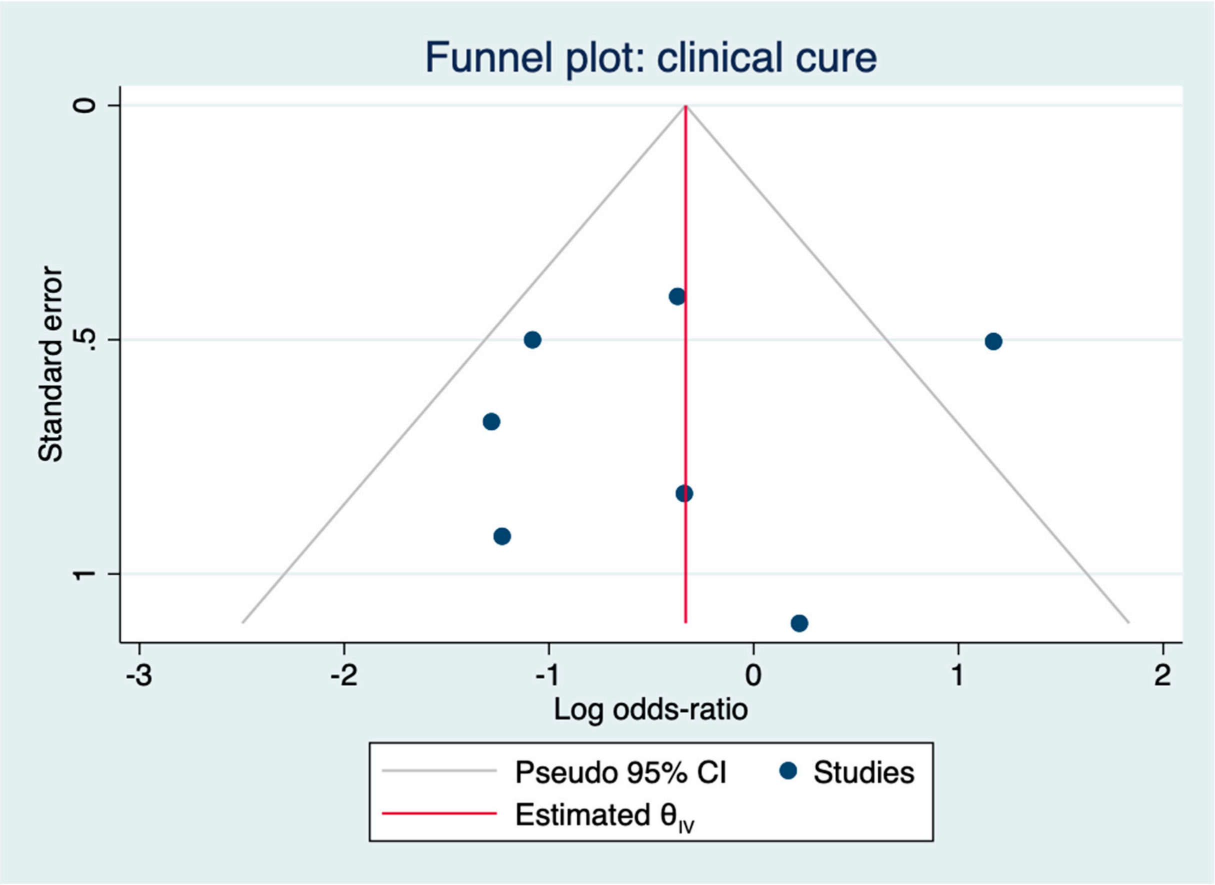

Supplementary Figure S1: Funnel plot of the publication bias for clinical cure.
